# Secretory breast carcinoma in a 12-year-old girl: A case report

**DOI:** 10.3892/ol.2014.2380

**Published:** 2014-07-24

**Authors:** YA-QI WANG, YU WANG, JING-HUA ZHANG, YU-FENG LI, HONG-MIN LI, LEI WANG, YUAN YAO

**Affiliations:** 1 Department of Breast Cancer Surgery, Tangshan People’s Hospital, Tangshan, Tangshan, Hebei 063001, P.R. China; 2 Graduate Department, Hebei United University, Tangshan, Tangshan, Hebei 063001, P.R. China; 3 Central Laboratory, Cancer Institute, Tangshan People’s Hospital, Tangshan, Tangshan, Hebei 063001, P.R. China; 4 Department of Pathology, Tangshan People’s Hospital, Tangshan, Tangshan, Hebei 063001, P.R. China; 5 Department of Gastrointestinal Tumor Surgery, Tangshan People’s Hospital, Tangshan, Tangshan, Hebei 063001, P.R. China; 6 Breast Disease Prevention and Control Center, Tangshan, Tangshan, Hebei 063001, P.R. China

**Keywords:** juvenile/secretory breast cancer, breast-conserving therapy, triple-negative carcinoma, a biopsy of the sentinel lymph node

## Abstract

Secretory breast carcinoma (SBC) is a rare tumor that was originally described in children and adolescent women, with a characteristic morphology and controversy regarding the choice of treatment. This unusual breast cancer subtype generally has a favorable prognosis, although several cases have been described in adults with increased tumor aggressiveness and a risk of metastases. Surgery is considered the most appropriate treatment for this pathology. The present study describes the case of a 12-year-old female who presented with a painless lump in the left breast, and subsequently underwent a biopsy of the sentinel lymph node and a partial resection of the left breast (breast-conserving therapy). Periodic follow-up examinations after completion of the surgical and chemotherapeutic treatment have shown no evidence of either local regression or distant metastases and, one year later, the patient remains free of the disease. This study suggests that local excision with sentinel lymph node mapping may be a suitable therapeutic approach for children with SBC

## Introduction

Secretory breast carcinoma (SBC) is one of the rarest types of breast cancer, accounting for <1% of all breast cancers. It was first reported as ‘juvenile breast carcinoma’ by McDivitt and Stewart in 1966 (1). Subsequently, additional cases in children (2–6) and adults (7–12) have been described. The original term was replaced by the more appropriate ‘secretory breast cancer’ in the 1980s.

The typical clinical presentation of SBC is a slow-growing, painless, well-circumscribed, mobile, palpable mass occurring anywhere in the breast. SBC ultrasound appearance shows a solitary, microlobulated, hypoechoic mass resembling a benign lesion, such as a fibroadenoma or other well-circumscribed carcinomas (13). There are, at present, no consensus guidelines for the treatment of SBC. Although recommendations vary among authors, surgical excision is the primary mode of treatment for secretory carcinoma. However, preservation of pubertal breast tissue is important to ensure correct breast development; although, this is not always possible due to the location of the tumor. Occasionally, mastectomy is required, and this may cause complex psychosocial difficulties in the lives of adolescent females (14,15). The present study describes the case of a 12-year-old female with SBC and highlights the treatment options available for children with this tumor type. Patient provided written informed consent.

## Case report

**Figure 1:**
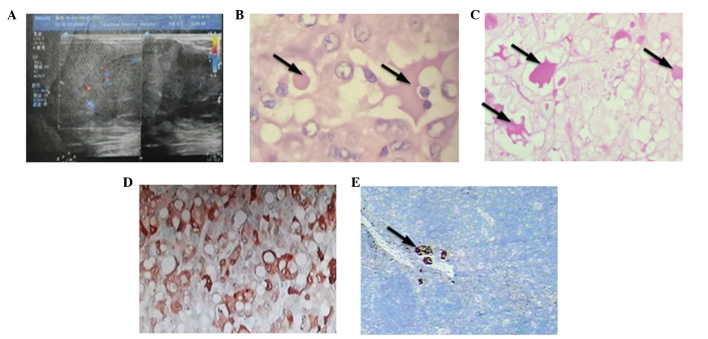
(A) Ultrasound examination of the breast shows a well-defined hypoechoic nodule, 4.0 cm in diameter, without calcification or evidence of invasion. (B) Photomicrograph shows cells arranged in a glandular pattern with eosinophilic secretion (arrows) within the lumen (hematoxylin and eosin staining; magnification, ×400). (C) The secretions (arrows) are strongly periodic acid-Schiff-positive (magnification, ×400) (arrows). (D) Positive immunostaining for S-100 (magnification, ×100). (E) The sentinel lymph node contains isolated tumor cells (arrows) (cytokeratin staining, magnification, ×100).

In June 2012, a 12-year-old female presented to the Department of Breast Cancer Surgery, Tangshan People’s Hospital (Tangshan, China) with a painless lump close to the left breast areola. The patient had first noticed the lump two months prior to presentation, during which time there had been no significant increase in the sixe of the lump. There was no history of local trauma, and no family history of relevant malignancy. Examination revealed a 4.0×3.0-cm, firm, well-circumscribed, mobile mass located eccentrically beside the areola at the 3 o’clock position. No nipple discharge or inversion were observed, and no clinical alterations were found in the ipsi- or contralateral lymph nodes. Ultrasound examination of the breast showed a well-defined hypoechoic nodule measuring 4.0×3.9×2.5 cm in size, without calcification or evidence of invasion (Fig. 1A). The mass was considered as benign in appearance and diagnosed as a fibroadenoma of the breast.

Not only for this reason, but also at the patient’s request, core biopsy of the tumor was not performed and the patient therefore immediately underwent surgical removal of the nodule under local anesthetic. The macroscopic examination of breast operative specimens revealed the presence of a nodule measuring 4.0×4.0×2.9 cm, and the lesion was void of an intact capsule. The cut surface appeared solid and gray-white. Microscopic analysis of hematoxylin and eosin-stained sections showed tumor cells arranged in lobules separated by dense fibrous septa (Fig. 1B). The lobules showed a tubuloalveolar and glandular pattern with abundant secretion, which is usually pale pink (16). A honeycomb pattern was also observed. The tumor consisted of cells with minimal to middle nuclear atypia with abundant slightly basophil cytoplasm, as well as intracytoplasmic vacuoles. The presence of secretions which are strongly periodic acid-Schiff-positive are a hallmark of SBC (Fig. 1C) (16). No nuclear division was observed in the present case. Immunohistochemistry was also performed, revealing that the tumor cells were negative for estrogen receptor (ER), progesterone receptor (PR) and human epidermal growth factor receptor-2, and showed positive staining for S100 (Fig. 1D). A diagnosis of secretory breast cancer was then established. Axillary sentinel lymph node (SLN) biopsy was also performed. The SLN was found to contain isolated tumor cells (Fig. 1E), and according to the NCCN Clinical Practice Guidelines in Oncology of Breast Cancer (17), cases of no metastases can be considered as SLN. Therefore, the patient could avoid complete axillary dissection.

Since the mass was close to the breast areola and the patient was younger than 35 years old, the patient seemed better to receive a radical mastectomy. However, the patient wished to undergo a partial resection for the tumor, rather than the radical mastectomy. The breast magnetic resonance imaging revealed no multiple lesions and the patient subsequently underwent a left breast conservative surgery. The histopathology of operative specimens revealed no evidence of residual neoplasia within the breast. After multidisciplinary consultation (including with a pediatric oncologist), adjuvant chemotherapy was administered to the patient. This comprised doxorubicin (60 mg/m^2^, IV) and cyclophosphamide (600 mg/m^2^, IV) on day one, cycled every 21 days for four cycles, followed by docetaxel (100 mg/m^2^, IV) on day one, cycled every 21 days for four cycles. No radiotherapy treatment was performed.

Periodic follow-up examinations after completion of the surgical and chemotherapeutic treatment have shown no evidence of either local regression or distant metastases to date and, one year later, the patient remains free of disease.

## Discussion

Since very few cases of SBC have been described in the literature, it is imperative to report any new cases observed in order to establish the most suitable therapeutic approach. SBC has a better prognosis than the more usual form of ductal carcinoma (18). There is no consensus of opinion as to how SBC should be treated, as there are so few case reports of SBC in the literature (19). However, surgery is the primary mode of treatment for this pathology. A previous study described the case of a 50-year-old woman who had undergone a breast conservative surgery for SBC (14). However, there are few case reports on conservative surgery treatment in adolescents, according to Costa’s study, as for children, local excision with sentinel lymph node mapping is the preferred initial treatment (14). This unusual breast cancer subtype generally has a favorable prognosis, although several cases have been described in adults with increased aggressiveness and a risk of metastases. The clinical course of SBC is characterized by a tendency for late local recurrence and prolonged survival, even with lymph node metastases, and mortality due to metastatic secretory carcinoma is extremely rare (20). The present study describes a case whereby breast-conserving therapy was used to treat secretory breast cancer in a 12-year-old female. The lack of secure data regarding the potential of secretory carcinoma to bring about local relapse leads us to the conclusion that a conventional, conservative approach, such as quadrantectomy followed by radiotherapy and/or chemotherapy, used for all other types of infiltrating breast carcinomas, should also be used for this type of tumor (20). There is insufficient evidence to recommend post-operative radiotherapy and/or chemotherapy for this pathology (9,21–23). However, in one case report, a patient with a single positive lymph node responded to traditional chemotherapy consisting of six cycles of cyclophosphamide, methotrexate and 5-fluorouracil, with no evidence of local or distant disease after seven years of follow-up (24). Although chemotherapy can cause potential reproductive risks, according to NCCN guidelines, even favourable histologies of breast cancer which are ER- and PR-negative should be treated as typical breast cancer. In the present case, considering the mass was 4.0 cm in diameter, adjuvant chemotherapy was administered, consisting of doxorubicin and cyclophophamide, followed by docetaxel. In consideration of no pronounced evidence demonstrates that ovarian suppression or other interventions decrease the toxicity of cytotoxic chemotherapy on the premenopausal ovary (25), no gonadotrophin-releasing hormone agonist treatment was administered. Postoperative radiotherapy should be proposed following conservative surgery in adult patients, but is not advised for children due to the possible secondary effects, such as fibrosis of the lung, rib damage and the consequent asymmetry of the rib cage, as well as a risk for future development of neoplasia (22,26). Due to this, no radiotherapy treatment was performed in the present case.

As for adolescents, fibroadenomata predominate (27); in the younger age group, more insidious pathology must be excluded. As studies in the literature have shown optimal results regarding the value of bioptic staging of the sentinel lymph gland (24,28,29), we believe that this treatment choice is particularly valid in order to avoid axillary lymphadenectomy. Given the uncertainty regarding the latency and dormancy of the tumor, close clinical follow-up of the present patient for an indefinite period was considered mandatory. We propose that it is useful to report every case of this rare tumor, in order to increase the knowledge of its biology and management.

## Abbreviations

SBC: secretory breast cancer
SLN: sentinel lymph node
ER: estrogen receptor
PR: progesterone receptor
